# Ultrafast and Sensitive Self‐Powered Photodetector Based on Graphene/Pentacene Single Crystal Heterostructure with Weak Light Detection Capacity

**DOI:** 10.1002/advs.202204332

**Published:** 2022-10-26

**Authors:** Yuquan Gan, Shuchao Qin, Qianqian Du, Yuting Zhang, Jing Zhao, Mengru Li, Anran Wang, Yunlong Liu, Shuhong Li, Ruixin Dong, Linglong Zhang, Xiaoqing Chen, Cailong Liu, Wenjun Wang, Fengqiu Wang

**Affiliations:** ^1^ Key Laboratory of Optical Communication Science and Technology of Shandong Province School of Physical Science and Information Engineering Liaocheng University Liaocheng 252059 China; ^2^ National Laboratory of Solid State Microstructures and Jiangsu Provincial Key Laboratory of Advanced Photonic and Electronic Materials School of Electronic Science and Engineering Nanjing University Nanjing 210093 China; ^3^ College of Physics Nanjing University of Aeronautics and Astronautics Key Laboratory of Aerospace Information Materials and Physics (NUAA) MIIT Nanjing 211106 China; ^4^ Key Laboratory of Light Field Manipulation and Information Acquisition Ministry of Industry and Information Technology and Shaanxi Key Laboratory of Optical Information Technology School of Physical Science and Technology Northwestern Polytechnical University Xi'an 710129 China

**Keywords:** heterostructures, pentacene single crystals, self‐powered photodetectors, ultrafast speed

## Abstract

Organic materials exhibit efficient light absorption and low‐temperature, large‐scale processability, and have stimulated enormous research efforts for next‐generation optoelectronics. While, high‐performance organic devices with fast speed and high responsivity still face intractable challenges, due to their intrinsic limitations including finite carrier mobility and high exciton binding energy. Here an ultrafast and highly sensitive broadband phototransistor is demonstrated by integrating high‐quality pentacene single crystal with monolayer graphene. Encouragingly, the −3 dB bandwidth can reach up to 26 kHz, which is a record‐speed for such sensitized organic phototransistors. Enormous absorption, long exciton diffusion length of pentacene crystal, and efficient interfacial charge transfer enable a high responsivity of >10^5^ A W^−1^ and specific detectivity of >10^11^ Jones. Moreover, self‐powered weak‐light detection is realized using a simple asymmetric configuration, and the obvious zero‐bias photoresponses can be displayed even under 750 nW cm^−2^ light intensity. Excellent response speed and photoresponsivity enable high‐speed image sensor capability in UV‐Vis ranges.  The results offer a practical strategy for constructing high‐performance self‐powered organic hybrid photodetectors, with strong applicability in wireless, weak‐light detection, and video‐frame‐rate imaging applications.

## Graphical abstract

An ultrafast and highly sensitive broadband phototransistor is demonstrated, with a record‐electronic bandwidths of ≈26 kHz for the sensitized organic phototransistors with high responsivity. The device with asymmetric configuration exhibit appreciable self‐powered weak‐light detection behavior under ultra‐weak light intensity.

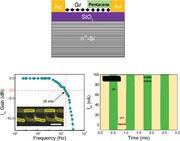

## 1 Introduction

Organic materials have attracted intense attention as a promising candidate for next generation electronics and optoelectronics, due to their advantages in electronic transport, strong light–matter interaction, low‐temperature processability, diversified bandgap selection, and intrinsic mechanical flexibility.^[^ ^1^, ^2^, ^3^, ^4^ ^]^ Enhancing carrier mobility and mitigating structural defects are crucial for organic electronics applications, and as such obtaining optimized molecular packing structures is actively pursued. In the last few decades, plethoric efforts have focused on improving the crystalline quality and developing new growth methods, optimizing device configuration and interfacial contact, etc., and encouraging results in high‐performance nanoelectronic devices have been achieved.^[^ ^5^, ^6^, ^7^, ^8^, ^9^, ^10^ ^]^

For optoelectronic applications, organic materials are often incorporated into other hybrid structures comprising of amorphous or polycrystalline films. However, extrinsic defects and grain boundaries in amorphous or polycrystalline films degrade the electron migration and/or exciton manipulation inside the semiconductors, so that high‐performance organic photodetectors are still facing severe challenges. For photodetectors employing organic crystals, only modest responsivity enhancement is demonstrated, which are limited by their finite carrier mobility (<50 cm^2^ V^−1^ s^−1^) and strong Coulomb interactions due to low dielectric constant.^[^ ^11^, ^12^, ^13^ ^]^

An effective strategy is integrating organic materials with high mobility materials such as graphene to enhance their photoresponse using the photogating effect.^[^ ^14^, ^15^, ^16^, ^17^, ^18^, ^19^, ^20^ ^]^ In these systems, organic material absorbs photons and produces electron–hole pairs, then one type of carriers will be captured to form a localized field. The photogenerated carrier recombination time is significantly prolonged by photo‐induced interfacial localized field, resulting in a high gain (*G* = *τ*_life_/*τ*_transit_). In the past few years, photodetectors with high responsivity have been demonstrated primarily by employing different organic films. Unfortunately, high density of trap states and inhomogeneity in organic films severely limits the response speed. Short exciton diffusion lengths (5–10 nm) of amorphous films due to the high defect density confine the efficient photon–electron conversion near the hetero‐interface region,^[^ ^21^ ^]^ which seriously limits the quantum efficiency. In addition, it is typically very hard to operate such sensitized photodetectors under zero bias, due to the mirror symmetric potential profile at the metal–graphene junctions, making it not applicable for the emerging applications in wireless sensor networks and wearable medical monitoring systems. Dual‐gate structure, asymmetric metal design, and tunneling layer engineering have been demonstrated for self‐powered photodetector, but all these strategies require complicated fabrication and modulation processes.

In this study, we demonstrate an ultrafast and highly sensitive photodetector based on pentacene crystal/graphene heterostructure, over the ultraviolet to the near‐infrared range. The sensitized device shows a record‐level rise/decay time of ≈6/9 µs, which should be attributed to the low charge trap density in the crystal and pentacene/graphene interface as well as the short carrier lifetime in pentacene. Salient absorption and long‐range herringbone stacking of pentacene crystal significantly facilitate the diffusion of excitons toward the pentacene/graphene interface, enabling a high responsivity of 10^5^ A W^−1^ and a specific detectivity of >10^11^ Jones at a wavelength of 658 nm. Moreover, a zero‐biased photoresponse is observed in this sensitized system, and this self‐powered behavior is prominently enhanced by using a simple, asymmetric coupling design. More importantly, the device exhibit appreciable zero‐biased photoresponses even under an ultra‐weak light intensity of ≈750 nW cm^−2^. Finally, several high‐resolution single pixel imagings in the UV and the visible ranges were demonstrated, utilizing their fast speed and high responsivity. These results suggest that organic single crystal‐based sensitized self‐powered device with high photodetection capability possess enormous potential in wireless broadband photodetectors for high‐frequency, weak‐light detection.

## 2 Results and Discussion

**Figure 1:**
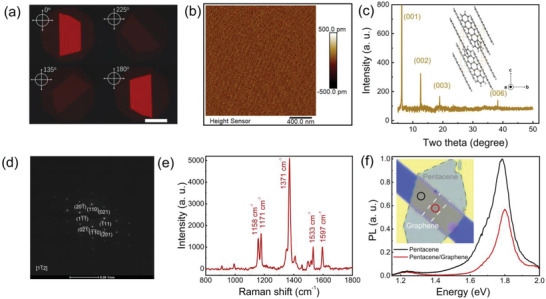
Characterization of the pentacene single crystal and its heterointerface. a) Cross‐polarized optical microscopy images of a pentacene single crystal in different polarization directions. Scale bar: 70 µm. b) The surface morphology of a pentacene single crystal. c) XRD pattern of the pentacene single crystal. d) Selected‐area electron diffraction (SAED) pattern of pentacene single crystal depicting zone axes of [1$\bar{1}$2]. e) Raman shift of pentacene single crystal in the high frequency region. f) Photoluminescence spectra of pentacene single crystals with (red) and without (black) graphene. Inset: Optical microscope image showing the test area as well as the graphene channel.

The pentacene single crystals in experiment were grown onto SiO_2_/Si substrate by the microspacing in‐air sublimation (see Figure S1, Supporting Information). To testify macroscopic molecular ordering and absence of polycrystalline domains, we used the previous well‐established technology of polarized optical microscopy (POM).^[^ ^22^ ^]^ **Figure** **1**a shows the POM images of the as‐fabricated pentacene crystal using a 625 nm LED as the light source. The pentacene single crystal exhibits the uniform brightness in entirety interface and shows obvious brightness variation dependent on the angle between the long‐axis of crystal and the polarization plane of incident light, indicating a pristine anisotropic single crystal structure. For pentacene single crystal, the surface morphology was investigated using atomic force microscopy (AFM), as shown in Figure 1b. The root‐mean‐square roughness is only 84 pm, indicating its atomic‐scale flatness. X‐ray diffraction (XRD) patterns of the single crystal were also investigated, as shown in Figure 1c. A series of diffraction peaks at low angle 2*θ* = 6.34°, 12.6°, 18.94°, and 38.28° are easily recognizable, corresponding to the (001), (002), (003), and (006) planes of triclinic single crystal, indicating that the *c*‐axis is vertical to the *ab* plane. The high crystallinity is also confirmed by the selected‐area electron diffraction in Figure 1d, which exhibits the well‐defined individual electron diffraction spots rather than circular lines. Raman scattering is an analytical method for reflecting the molecular vibration and rotation direction. We measured the Raman signal of pentacene crystal on graphene sheet. For graphene, Raman peaks are observed around the 1580 cm^−1^ (G‐band) and 2696 cm^−1^ (2D‐band) without D peak and the intensity of 2D‐band is almost twice that of G‐band, indicating a pristine monolayer graphene with reasonably good quality, as shown in Figure S2, Supporting Information. For the pentacene single crystal, a strong molecular vibrational peak at 1371 cm^−1^ and two weak peaks at 1533 and 1597 cm^−1^ belong to the benzene ring stretching, as shown in Figure 1e. The vibrational peaks at 1158 and 1171 cm^−1^ should be attributed to E_2g_ vibration of the benzene ring, which can be approximately described as C—H deformation. These Raman peaks are corresponding to the results of pentacene crystal.^[^ ^23^, ^24^ ^]^ In Figure 1f, the strong peak of photoluminescence (PL) spectra is observed at 1.8 eV, implying that the *c*‐axis is vertical to the crystal surface, as were determined from XRD and Raman results. The weak PL shoulder peak at 1.95 eV confirms a pure crystal with few deep trap states. Comparing the two PL spectra, the presence of graphene underneath pentacene causes the PL quenching (reduced by about 40%), indicating that a substantial fraction of excitons dissociate across the pentacene/graphene interface.

**Figure 2:**
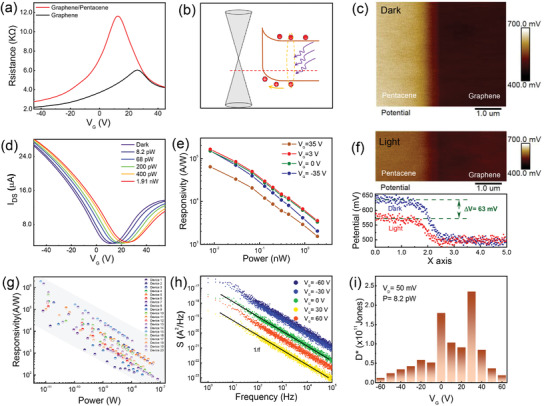
Optical gating in the pentacene–graphene interface. a) Transfer characteristic of pentacene single crystal/graphene transistor compared with that of pristine graphene transistor. b) Schematic illustration of charge transfer at pentacene single crystal/graphene interface. c) KPFM image of pentacene and graphene interface under dark condition. d) Transfer curves of pentacene single crystal/graphene transistor under different optical powers (*V*_D_ = 50 mV, *λ* = 658 nm). e) Responsivities as a function of light powers under different gate voltages. f) KPFM image (Above) of pentacene and graphene interface under illumination, and the potential profile difference (Bottom) under light and dark conditions. g) Photoresponse statistics for other fabricated 20 hybrid devices. h) Noise current spectral density as a function of frequency under different gate bias. i) The specific detectivity (D*) as a function of gate voltage (*V*_G_) at finite illumination (*λ* = 658 nm, *P* = 8.2 pW).

Transfer curves of the pristine graphene transistor and the graphene/pentacene device are compared in **Figure** **2**a. It is observed that Dirac point of the transistor shifted from 25 to 12 V after covering pentacene single crystal, indicating an n‐type doping (electron‐doping) for graphene sheet by the pentacene layer. Based on the equation *n* = Δ*V* × *C*_g_/*e*, in which *C*_g_ is capacitance per unit area, we estimate that electron‐doping level is on the order of ≈10^12^ cm^−2^. This electrostatic doping causes an upward bending in energy level of pentacene (including the highest occupied molecular orbital and the lowest unoccupied molecular orbital) near the graphene/pentacene junction in Figure 2b. The origin of energy bending is the higher Fermi of pentacene than that of graphene (≈4.7 eV).^[^ ^25^, ^26^ ^]^ The energy band barrier and charge transfer at the graphene/pentacene interface are further corroborated by Kelvin probe force microscopy (KPFM), as shown in Figure 2c (see in situ AFM surface topography in Figure S3, Supporting Information). The contact potential difference (CPD) between the AFM tip (Pt/Ir coated tips) and the sample is defined as:*V*_CPD_ = (*φ*_tip_ − *φ*_sample_)/*q*, where *φ*_tip_, *φ*_sample_, and *q* are the work functions of the tip, sample, and the elementary charge, respectively. From this equation, the difference value of *V*_CPD_ signal between the graphene and pentacene can be expressed as Δ*V*_CPD_ = *V*_Pentacene_ − *V*_Graphene_ = (*φ*_Graphene_ − *φ*_Pentacene_)/*q*, and it is about 130 mV, which indicates an energy band configuration for individual material system, as shown in Figure S4, Supporting Information. The strength of band bending can be also regulated by gate bias (see details in Figure S4, Supporting Information). Under illumination, this upward bending energy level would drive the photogenerated‐hole in pentacene into graphene, while electrons remain in the pentacene single crystal, causing the up‐shift of Dirac point in Figure 2d. The up‐shift of the Dirac point as a function of the illumination power is plotted in Figure S5, Supporting Information, clearly revealing its high sensitivity at low power density. According to the photocurrent (*I*_ph_ = |*I*_light_ − *I*_dark_|) measured at *V*_DS_ = 50 mV in Figure S6a, Supporting Information, we calculated the photoresponsivity (defined *R* = *I*_ph_/*P*_in_) of device for 658 nm light, as shown in Figure 2e. The *R* of our device can reach ≈10^5^ A W^−1^ under low light power at *V*_D_ = 50 mV, which is much higher than that of reported organic photodetector at similar excitation intensity.^[^ ^17^, ^27^, ^28^, ^29^, ^30^, ^31^, ^32^ ^]^ As with most graphene‐related phototransistors, *R* decreases at high incident illumination levels, which is caused by the saturated photogating effect, which can be understood in terms of three effects. One is the saturated absorption from the pentacene single crystal. The other one is the shorten life‐time of photoexcited carrier that is approximately expressed as *τ* = 1/*β*(*n*_e_ + *n*_h_ + Δ*n*_e_), where *β* is the recombination probability of nonequilibrium carrier under illumination. Moreover, high photoexcited carrier densitiesΔ*n*_e_ would increase the recombination probability. According to expecting theory, the built‐in field should be also weakened at the pentacene/graphene interface in light condition. To further verify the variation of built‐in field, we carried out KPFM under a 658 nm laser irradiation, as shown in Figure 2f. Compared with the value in dark, the difference value of *V*_CPD_ signal between the graphene and pentacene decreased by ≈50%, reflecting the key role of built‐in field for the highly sensitivity. At large gate voltages, the responsivity and photocurrent dramatically diminished (Figure 2e and Figure S6a, Supporting Information), which is related to higher extrinsic carrier density in graphene. In addition, the linear scaling of the photocurrent with the bias voltage is clearly observed in Figure S6b, Supporting Information, implying that we can obtain a higher responsivity under a high source–drain voltage. To verify the universality of our devices for high responsivity, we made a statistic of the responsivity for other fabricated 20 devices. The results indicate that 85% of devices can achieve high responsivity of 10^5^ A W^−1^, and even 10% of devices can achieve up to 10^6^ A W^−1^ at low optical power, as shown in Figure 2g (see details in Figure S7, Supporting Information). The specific detectivity (D*, units cm Hz^1/2^ W^−1^) is a significant indicator for the high‐performance photodetector, which reflects the signal to noise ratio and the sensitivity of device. Based on noise equivalent power $\mathrm{NEP}=(\sqrt{\int_{0}^{\Delta f}S\left(f\right)\mathrm{df}/\Delta f})/R$, D* can be calculated as $D*=\frac{A^{1/2}}{\mathrm{NEP}}\approx \frac{RA^{1/2}}{S{\left(f\right)}^{1/2}}$ at a small measurement bandwidth Δ*f* = 1 Hz, where *A* is the device area. Figure 2h shows the noise density spectral of the device as a function of frequency under different gate bias. All noise spectra at whole operated frequency exhibit a typical 1/*f* power density, indicating that the noise is mainly dominated by the fluctuations of carrier density or mobility. Using the experimental noise density spectra, we calculated that the value of D* can reach to 2.4 × 10^11^ Jones at *V*_G_ = 30 V (Figure 2i).

**Figure 3:**
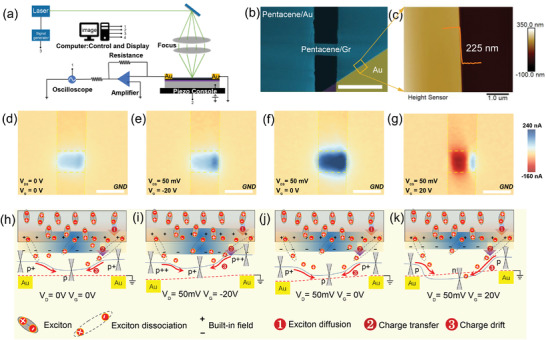
Photocurrent generation in pentacene single crystal/graphene symmetric device. a) Schematic diagram of photocurrent mapping set‐up with a laser wavelength of 658 nm, spot diameter of *d* ≈1 µm, and modulated frequency of 1.5 kHz. b) False‐color scanning electron microscopy (SEM) image of the device. Scale bar: 30 µm. c) AFM image corresponding to the marked positions in (b). d–g) Scanning photocurrent images measured at zero bias and different gate biases. Scale bar : 15 µm. h–k) Schematic band diagrams of the device at zero bias and different gate biases. A couple of red circles marked “+”and “−” indicate the un‐dissociated excitons. They can be dissociated under built‐in electric field (black “+”and “−” symbol) after diffusing into the interface from the pentacene bulk, and transferring to the graphene forming the photocurrent.

To further verify the photocurrent generation mechanism in our hybrid device, high‐resolution spatial photocurrent mapping was employed under a confocal optical microscope. **Figure** **3**a shows the schematic of the photocurrent measurement set‐up with a laser wavelength of 658 nm. Figure 3b shows the false‐color scanning electron microscopy (SEM) image of measuring hybrid device. Here, the thickness of pentacene single crystal is about 225 nm, as shown in Figure 3c. A sequence of scanning photocurrent images taken under fixed bias (*V*_D_ = 0 V or 50 mV) and different values of gate voltages are shown in Figure 3d–g. Scanning photocurrent images show the clearly current signals within the interior of the whole hybrid channel, indicating that the key principle is dominated by photogating (photoconductive) effects. Interestingly, the device exhibits an obvious zero‐biased photocurrent signal (Figure 3d), which should be origin from the photovoltaic effect contribution. Generally, because of the inhomogeneous doping of graphene, the device would form into the two asymmetry graphene/Au contact barriers. This physical mechanism is also verified by the inhomogeneous photocurrents in Figure 3e,f, where the device exhibits the slightly higher photocurrent near the right electrode/channel junctions than that of the middle of channel. As shown in Figure 3e–g, under fixed source–drain bias of 50 mV, the gate bias can directly modulate the amplitude, position, and direction of the photocurrent, which can be explained by using the band diagrams under different gate voltages in Figure 3h–k. Under the illumination, a great deal of excitons (electron–hole pairs) are generated in pentacene single crystal, then they diffuse into the graphene/pentacene interface (Step 1) separating into the free carriers near the pentacene/graphene interface. The photogenerated holes transfer into the graphene channel (Step 2) under the built‐in electric field, forming the photocurrent with the help of contact barrier or external electric field (Step 3). For this reason, we observed distinguishable photocurrent under zero‐bias in this unstudied configuration, as shown in Figure 3h. Generally, graphene is p‐doping, so we can observe a positive photocurrent at zero or navigate gate biases (see Figure 3d–f). When the Fermi level of graphene is swept from p‐type to n‐type, the photocurrent in center channel would switch in the opposite sign (Figure 3g), retaining an unchanged sign near the right Au electrodes. Due to the difference of work function between Au and graphene (see Figure S8, Supporting Information), the graphene under the Au metal is hole‐doping, forming a p^+^–p junction at zero gate bias in Figure 3j. With negative *V*_G_, the work function of the graphene increases and so does the p^++^–p^+^ homojunction near the metal–graphene interface, which reduce the band slope to further suppress the electron–hole separation. On the other hand, in the positive *V*_G_ regime the middle‐graphene is tuned into n‐doping while graphene near the metal contacts remains p‐type case owing to charge pinning effects, forming a p‐n‐p type conduction regime. The photogenerated holes transferred would lead to a negative photocurrent in center and positive photocurrent near the metal electrodes (Figure 3g). Here the disappeared photocurrent in left Au–graphene junction should be ascribed to the source–drain bias offset, and the apparent photocurrent is measured at smaller source–drain voltage, as shown in Figure S9, Supporting Information. In this case, n‐type body of the graphene increases the band slope, promoting the electron–hole separation and enhancing the photocurrent near the electrodes.

**Figure 4:**
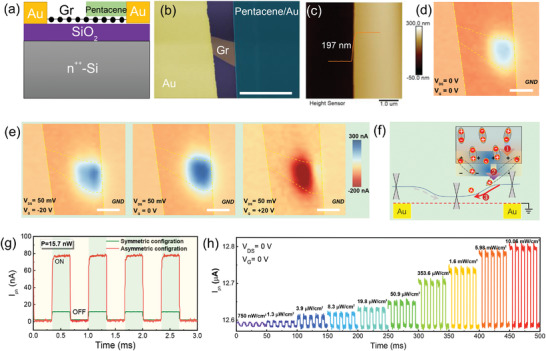
Photoelectrical characteristics of the asymmetric device. a) Structure diagram of the asymmetric device, in which the only half of graphene channel is covered by pentacene single crystal. b) False‐color scanning electron microscopy (SEM) image of the device. Scale bar: 20 µm. c) The height of the pentacene single crystal used in this device. d) Scanning photocurrent images measured at zero bias. Scale bar: 5 µm. e) Scanning photocurrent images measured under 50 mV bias with different gate voltages. f) Band diagram of photocurrent generation in this asymmetric configuration. g) Comparison of photocurrents in asymmetric and symmetric configurations under 15.7 nW light power (658 nm). h) Time‐resolved zero‐bias photoresponse of the device under different power intensities.

In most cases, the net photocurrent at zero‐bias in this symmetric configuration is very faint due to the mirror potential profile,^[^ ^33^, ^34^ ^]^ when the whole device exposures to the light. To enhance the self‐powered performance, we constructed an asymmetric contact geometry that breaks the mirror offset phenomenon, as shown in **Figure** **4**a. Figure 4b shows the false‐color SEM image of the asymmetrical device where only half of graphene channel is covered by pentacene single crystal. The thickness of pentacene single crystal is about 197 nm, as shown in Figure 4c. With the assistance of photogating effect, the zero‐bias photocurrent clearly arises from the graphene/pentacene overlapping area in Figure 4d, indicating also the key role of pentacene single crystal for the photoresponse. The photocurrent sign under the drain bias can be consistently modulated by the gate bias, as shown in Figure 4e, indicating the cooperated contribution of photovoltaic and photoconductive effects. Other half‐covered device exhibits also the similar trends (see Figure S10, Supporting Information), implying the universality of this asymmetric design for the self‐powered characteristics. Compared with the symmetric device in which the graphene channel is fully covered by pentacene crystal, the photocurrent of this asymmetric configuration under zero source–drain bias is dramatically increased by about 650%, as shown in Figure 4g. This phenomenon can be explained by the band diagram in Figure 4f. When the incident light illuminates the whole device, the net short‐circuit current is given by *I*_ph_ = *I*_ph(R)_ − *I*_ph(L)_ = *J*_sc_ × *W*_R_ − *J*_sc_ × *W*_L_, where *J*_sc_ is the short‐circuit current density, and *W*_R_ and *W*_L_ are the contact lengths of the right and left graphene/pentacene junctions. For this asymmetric configuration, the photocurrent of left junction is nearly zero due to destitute pentacene element, resulting in an obvious net photocurrent. Weak light detection especially under zero bias is of significance in various applications, such as military monitoring and astronomical photography. To assess the weak light detection ability of our device, we carried out zero‐bias transient photoresponse. The recognizable and reliable optical switching capability of device was obtained even under extremely low light intensity of ≈750 nW cm^−2^ in Figure 4h, indicating its potential for the wireless biomedical imaging and military monitoring in future.

**Figure 5:**
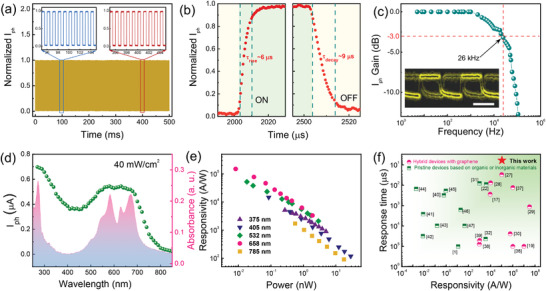
Performance metrics of pentacene single crystal/graphene device. a) Normalized temporal photocurrent response of the device at atmosphere environment (*V*_G_ = 0, *V*_DS_ = 50 mV, *λ* = 532 nm). b) The enlarged rise and decay edges of one cycle. c) Photocurrent gain expressed in dB as a function of the increasing light‐modulate frequency. Inset shows the eye diagram acquired at 5 kbit s^−1^. Scale bar: 200 µs. d) The temporal photocurrent of the device for different incident wavelength, and the bottom shaded area corresponds to the light absorption of the pentacene single crystal. e) Responsivities as a function of optical power for several UV and visible laser diodes (375, 405, 532, 658, and 785 nm). f) Benchmark graph of responsivity (*R*) and response time (*τ*) in this work compared to previously reported advanced devices based on organic materials and hybrids, demonstrating its excellent overall performances.

To confirm the temporal photoresponse characteristic, the photocurrent with periodically switched illumination was measured under a small bias voltage of 50 mV. Such organic heterostructure device exhibits robust switching behavior and excellent reproducibility. Even at ambient condition, the device keeps a good responsivity without any degradation after 500 cycles for 532 nm illumination, as shown in **Figure** **5**a. Moreover, the device have excellent long‐term stability, which benefits from the remarkable stability of graphene and organic crystals,^[^ ^35^ ^]^ and they still exhibit reliable photoresponse after 4 months in air without nitrogen protection (Figure S11, Supporting Information). These results indicate that high quality organic crystal hybrids own excellent environmental robustness and reliability, which are critical to their practical application. Response speed of the device is another key parameter for advanced photodetectors. The rise time (*τ*_r_) and decay time (*τ*_d_), are defined as the time required to increase from 10% to 90% of the maximum photocurrent and decrease from 90% to 10% of the maximum photocurrent. From the amplifying temporal photoresponse in Figure 5b, we see that the *τ*_r_ and *τ*_d_ of the device are estimated to be 6 and 9 µs, respectively. Under zero source–drain bias, the *τ*_r_ and *τ*_d_ of device are about 12 and 13 µs (Figure 4g). The photocurrent gain as a function of frequency is also measured, as shown in Figure 5c. The cutoff frequency at −3 dB, that is, at which the photocurrent signal is reduced by square root of 2, is measured at about 26 kHz at *V*_G_ = 0 V, which is one of the highest values among these reported organic photodetector.^[^ ^17^, ^19^, ^22^, ^27^, ^31^, ^36^ ^]^ Excellent −3 dB bandwidth of 26 kHz, coupled with extremely sensitive photodetection means that such hybrid organic devices have the great potential in video‐frame‐rate imaging applications. The eye diagram with an open eye at 5 kbit s^−1^ in inset of Figure 5c confirmed the feasibility of high‐speed imaging. To access the capability of such organic hybrid device as the broadband photodetectors, we measured the broadband photoresponse characteristics from UV to NIR range (270–850 nm) in Figure 5d. Here two responsivity peaks are achieved at UV and visible range, which is consistent with the absorption of pentacene single crystals, as shown in shaded areas in Figure 5d. From the responsivity trend, we can calculate that such hybrid device can achieve the higher photoresponse for <300 nm light illumination. For this sensitized phototransistor, the external quantum efficiency (EQE) can be calculated by the equation:$\mathrm{EQE}=\frac{hc}{\lambda q}\times \frac{I_{\mathrm{ph}}}{P_{\mathrm{in}}}\times \frac{L^{2}}{\mu V_{d}\tau_{\mathrm{decay}}}$, where *h*, *q*, and *λ* are Planck's constant, elementary charge, and the wavelength, respectively. Here the mobility *μ* is about 3510 cm^2^ V^−1^ s^−1^. According the photocurrent‐wavelength relation in Figure 5d, the EQE value of the device for the visible photon is about 3.3% without gain, and it can reach up to 10% at UV range, as shown in Figure S12, Supporting Information. In addition, the responsivity‐power is measured by using several UV, visible, and near‐infrared laser diodes in Figure 5e. At the illumination power of ≈1 nW, all wavelengths exhibit a responsivity > 10^3^ A W^−1^. To evaluate the performance index of our device, we compared previous advanced hybrid photodetector in recent years in Figure 5f.^[^ ^37^, ^38^, ^39^, ^40^, ^41^, ^42^, ^43^, ^44^, ^45^, ^46^, ^47^ ^]^ The performances of most devices exhibit a clear trade‐off between their responsivity and response speed. And the direction of the arrow represents the trend of idealization and optimization. Compared with those of reported photodetector, our device exhibits the better overall performances with the balance of photoresponsivity and time response.

**Figure 6:**
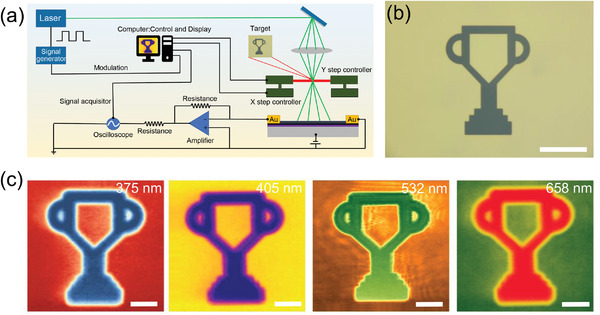
High‐speed imaging applications. a) Schematic diagram of the single pixel imaging measurement system. The modulated laser is focused onto the device by an objective lens. During the measurements, the measured object is moved by the step controller. b) Optical microscope image of the object under test. Scale bar: 40 µm. c) Acquired photocurrent imaging results of a trophy‐like object under different laser wavelengths (375, 405, 532, and 658 nm) at 1000 Hz modulated frequency. Scale bar: 20 µm.

In today's information age, high‐speed imaging plays a critical role in civil and military applications such as medicine diagnosis, environmental monitoring, space exploration, and enemy surveillance. To exploit the practical capability in video‐frame‐rate imaging from UV to visible range, we implemented several single‐pixel imaging employing 375, 405, 532, and 658 nm lasers by a home‐built system, as illustrated in **Figure** **6**a. The high frequency modulated light was fixed onto our device, and a patterned imaging target in Figure 6b located at the focal point was selected to modulate the light power, resulting in the change of photocurrent. The image consisting of 100 × 100 scanned points was formed by controlling the movement of a 2D control platform and recording the value of photocurrent. Figure 6c showed the corresponding high‐resolution trophy‐like images for 1000 Hz modulated UV and visible light illumination. The results indicate that our device is already capable for the broadband high‐resolution video‐frame‐rate imaging.

## 3 Conclusion

In summary, we successfully fabricated an ultrafast and highly sensitive broadband photodetector employing the pentacene single crystal and monolayer graphene. The photogating effect has been found to be the dominant photocurrent generation mechanism, resulting in a high responsivity of 10^5^ A W^−1^ and a specific detectivity of >10^11^ Jones at room temperature. Amazingly, the device exhibits an ultrahigh measuring −3 dB bandwidth of 26 kHz without any external auxiliary strategy, which is a record‐level for the current sensitized devices. More encouragingly, we realized the zero‐bias self‐powered weak‐light detection behavior under ultra‐weak light intensity as low as 0.75 µW cm^−2^ in a simple asymmetric configuration. Excellent response speed and sensitive photon detection enable its high‐speed image sensor capability, and several high‐resolution single pixel images are demonstrated as their imaging functionality. These results not only provided a strategy for constructing high‐performance self‐powered photodetectors, but also demonstrated that organic crystal hybrid device is a promising potential configuration in wireless, weak light detection, and video‐frame‐rate imaging applications in future.

## 4 Experimental Section

### Device Fabrication and Characterization

Pentacene powder (purity of 99.9%) was purchased from a commercial supplier (Alfa Aesar). The pentacene single crystal was grown onto SiO_2_/Si substrate by the microspacing in‐air sublimation. The growth temperature and time were 230 °C for 10 min. First, the graphene was mechanically exfoliated from natural crystals to the SiO_2_/Si wafer by the adhesive tape. Raman spectroscopy combined with optical microscope characterizations point to a defect‐free single‐layer sample. Then two Au patches with a thickness of 180 nm were mechanically transferred onto graphene sheet with a probe tip under the optical microscope, as the source/drain contact electrodes, to form a graphene FET. Finally, pentacene single crystals were overlapped onto the prefabricated graphene channel by dry transfer process.

The cross‐POM images were obtained by the Zeiss Imager A2m fluorescence microscopy. AFM measurements were performed using a Bruker Icon AFM operating at room temperature and ambient conditions. Raman and PL measurements were performed using a 532 nm excitation laser, ×100 objective lens with about 1 µm diameter spot size. Optical absorption spectrum was measured using a Craic 20/30 PV microspectroscopy. XRD of the crystal were performed by Panalytical X'pert3 MRD with a Cu‐K*α* anode operating at 40 kV and 40 mA.

### Electrical and Photoresponse Measurements

Electrical measurements were carried out by the Keithley 4200 parameter analyzer and Keithley 6482 at room temperature and ambient conditions. For photoresponse characterization, 375, 405, 532, 658, and 785 nm laser diodes were, respectively, used. Photocurrent mapping was performed under 658 nm laser illumination which was modulated by a square‐wave signal generator source. The incident light intensity was recorded by a power meter (Thorlabs PM100D). The fast temporal photoresponses of the device were recorded by a home‐build setup employing a high‐frequency oscilloscope and a low‐noise current preamplifier (Stanford Research SR570). Spectra responsivity was performed using a Newport Xenon lamp source and spectrophotometer. The noise current of device were recorded by a FS‐Pro Semiconductor parameter tester (Primarius Technologies Co., Ltd., China).

## Conflict of Interest

The authors declare no conflict of interest.

## Author Contributions

S.Q., Q.D., and F.W. conceptualized the idea and designed the phototransistor device. Y.G. fabricated the device and performed the experiments and initial data analysis. Y.Z. performed noise current spectral density measurement, AFM measurement, and discussed the photoresponsivity of device. J.Z. and M.L. fabricated the graphene sheets and pentacene crystals. A.W. performed the Raman and PL measurements and analysis. Y.L., S.L., and R.D. assisted with absorption and photoresponse measurements. L.Z., X.C., and C.L. contributed to data analysis. S.Q. and W.W. supervised the project and analyzed data. S.Q. and Q.D. drafted the manuscript and all authors contributed to the interpretation of results and assisted with preparation of the manuscript.

## Supporting information

Supporting Information

## Data Availability

The data that support the findings of this study are available from the corresponding author upon reasonable request.
