# Supplementary material for: Ultrafast and Sensitive Self‐Powered Photodetector Based on Graphene/Pentacene Single Crystal Heterostructure with Weak Light Detection Capacity
Source: Adv Sci (Weinh). 2022 Oct 26;9(35):2204332. doi: 10.1002/advs.202204332 (PMC9762291; doi:10.1002/advs.202204332)
Supplement: Supplementary file 1 — Supporting Information [file ADVS-9-2204332-s001.pdf]

ADVANCED SCIENCE
Open Access

Supporting Information

for *Adv. Sci.*, DOI 10.1002/advs.202204332

Ultrafast and Sensitive Self-Powered Photodetector Based on Graphene/Pentacene Single Crystal Heterostructure with Weak Light Detection Capacity

Yuquan Gan, Shuchao Qin, Qianqian Du*, Yuting Zhang, Jing Zhao, Mengru Li, Anran Wang, Yunlong Liu, Shuhong Li, Ruixin Dong, Linglong Zhang, Xiaoqing Chen, Cailong Liu, Wenjun Wang* and Fengqiu Wang**

Supporting Information

Ultrafast and Sensitive Self-Powered Photodetector based on Graphene/Pentacene Single Crystal Heterostructure with Weak Light Detection Capacity

Yuquan Gan, Shuchao Qin, Qianqian Du*, Yuting Zhang, Jing Zhao, Mengru Li, Anran Wang, Yunlong Liu, Shuhong Li, Ruixin Dong, Linglong Zhang, Xiaoqing Chen, Cailong Liu, Wenjun Wang*, and Fengqiu Wang**

Y. Q. Gan, S. C. Qin, Q. Q. Du, Y. T. Zhang, J. Zhao, M. R. Li, Y. L. Liu, S. H. Li, Prof. R. X. Dong, Prof. C. L. Liu, Prof. W. J. Wang
Key Laboratory of Optical Communication Science and Technology of Shandong Province, School of Physical Science and Information Engineering, Liaocheng University, Liaocheng 252059, China
E-mail: lcqinshuchao@126.com; dzdq0126@163.com; phywwang@163.com

A. R. Wang, Prof. F. Q. Wang
National Laboratory of Solid State Microstructures and Jiangsu Provincial Key Laboratory of Advanced Photonic and Electronic Materials, School of Electronic Science and Engineering, Nanjing University, Nanjing 210093, China
E-mail: fwang@nju.edu.cn

L. L. Zhang
College of Physics, Nanjing University of Aeronautics and Astronautics, Key Laboratory of Aerospace Information Materials and Physics (NUAA), MIIT, Nanjing 211106, China.

X. Q. Chen
Key Laboratory of Light Field Manipulation and Information Acquisition, Ministry of Industry and Information Technology, and Shaanxi Key Laboratory of Optical Information Technology, School of Physical Science and Technology, Northwestern Polytechnical University, Xi'an 710129, China

Keywords: Pentacene Single Crystal, Heterostructure, Ultrafast Speed, Self-Powered Photodetector

contents

S1. Pentacene single crystals

S2. High quality graphene film

S3. KPFM images of pentacene/graphene interface

S4. Schematic band diagrams between pentacene and graphene

S5. Dirac point shift of the device

S6. Photocurrent of the device

S7. Universality of responsivity

S8. KPFM images of Au/graphene

S9. Photocurrent mapping at V_{DS}= 5 mV

S10. Photocurrent mapping of another device with asymmetric configuration

S11. Durability of the device

S12. External quantum efficiency

S1. Pentacene single crystals

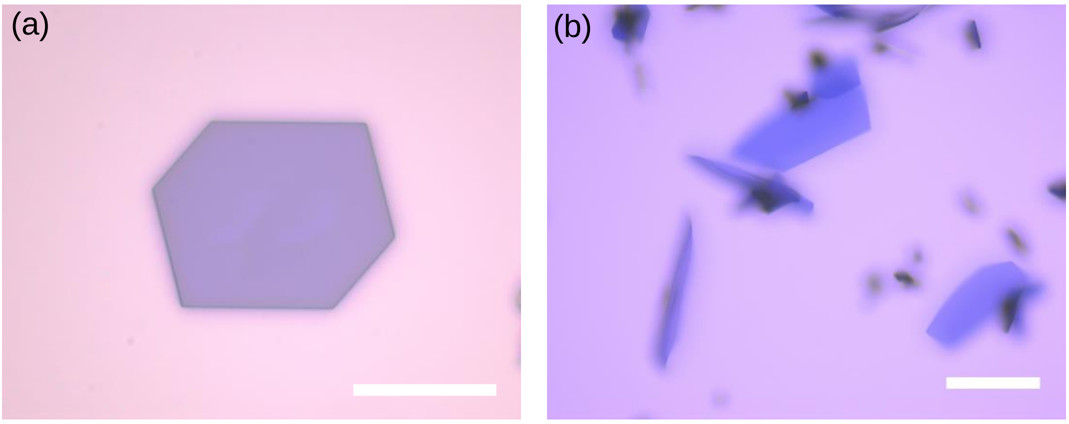

Figure S1. (a) The as-fabricated pentacene single crystal horizontally onto SiO_2/Si substrate. Scale bar: 35 µm. (b) The standing pentacene single crystal on the substrate. Scale bar: 50 µm.

S2. High quality graphene film

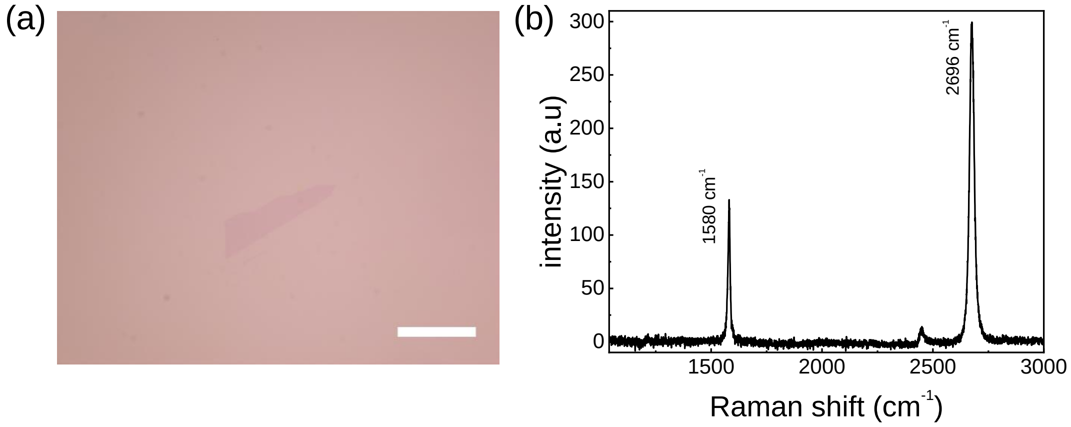

Figure S2. (a) Optical microscopy images of a graphene sheet. Scale bar: 15 µm. (b) Raman spectroscopy of the graphene sheet.

S3. KPFM images of pentacene/graphene interface

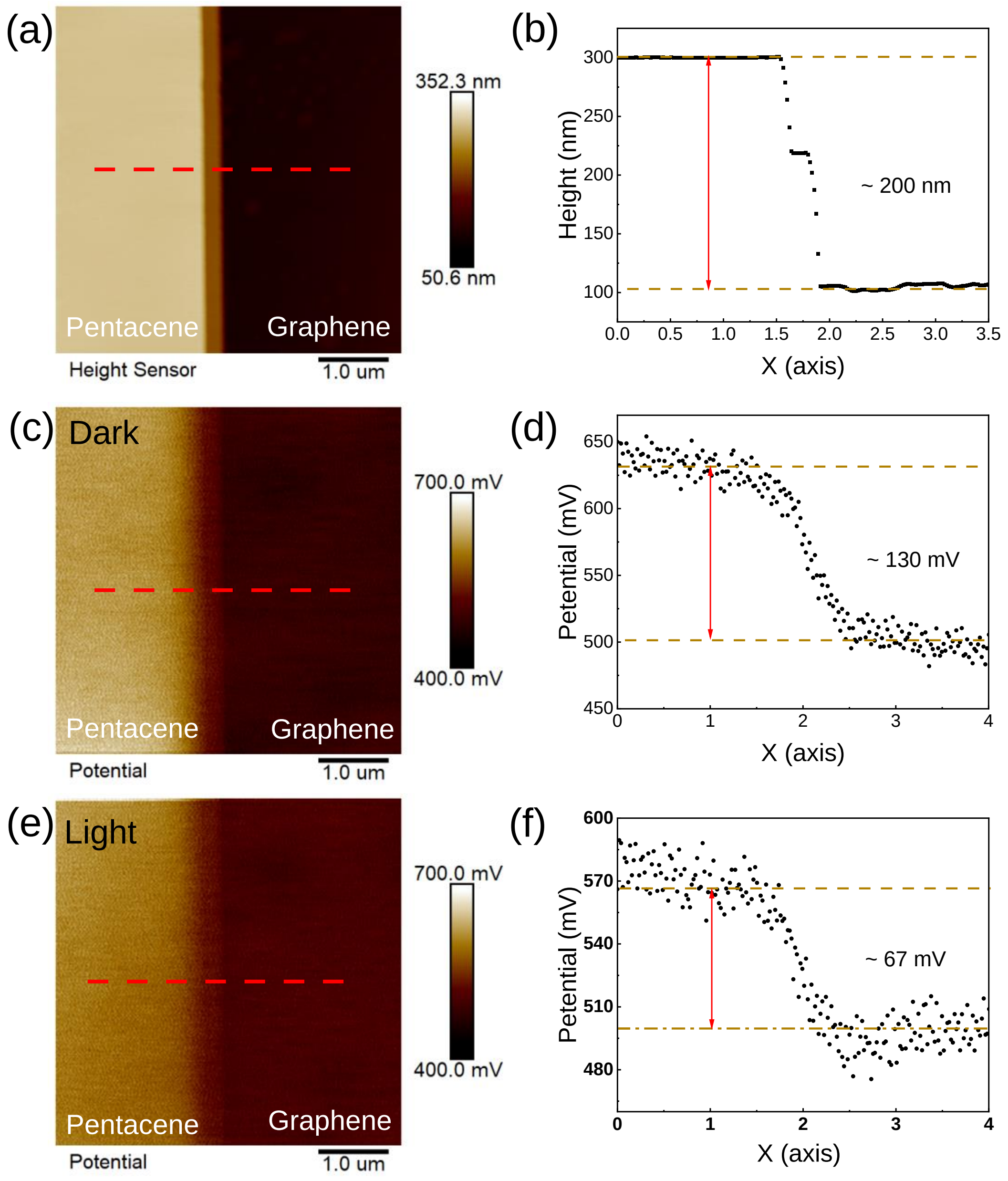

Figure S3. (a) AFM image of pentacene/graphene interfaces. (b) Height profile image of the interface between pentacene and graphene. (c) KPFM image in dark. (d) Potential profile under dark. (e) The corresponding KPFM image under light illumination (λ = 658 nm). (f) Potential profile under light illumination.

S4. Schematic band diagrams between pentacene and graphene

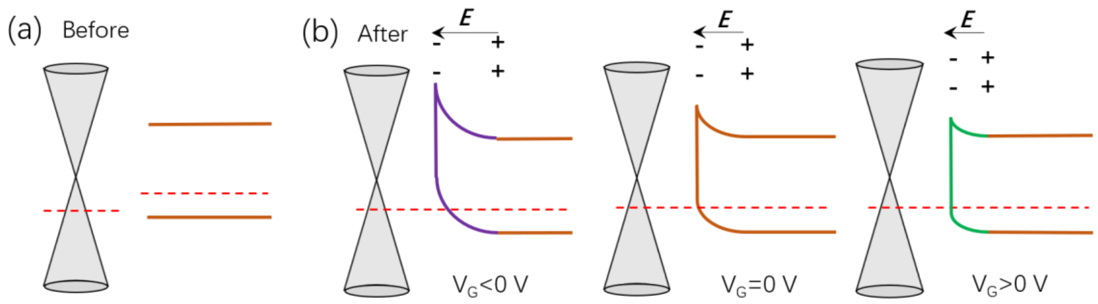

Figure S4. (a) Energy profile of individual pentacene and graphene. (b) Schematic band diagram after contacting each other under different gate voltages. Red dashed lines indicate Fermi level for hybrid system, and E indicate the built-in field.

S5. Dirac point shift of the device

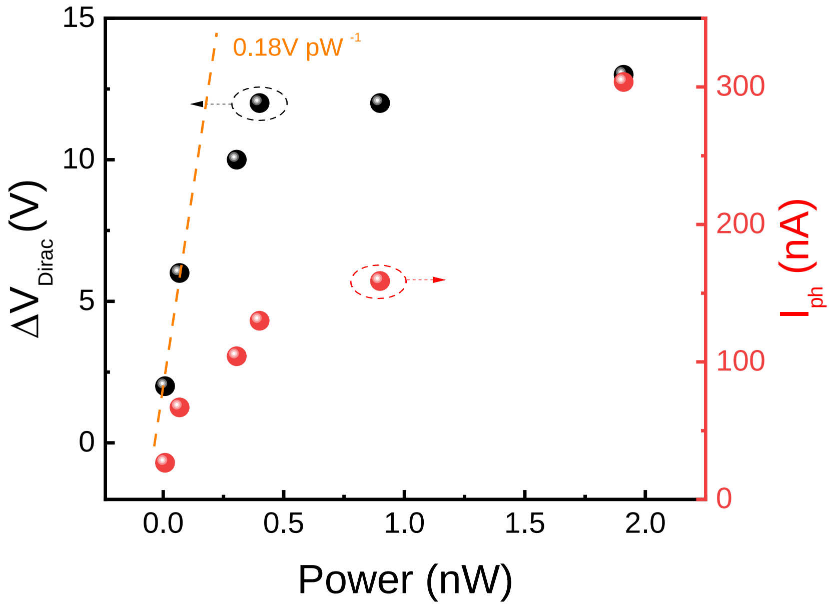

Figure S5. Shift of Dirac point as a function of the illumination power. With the increase of light intensity, the built-in electric field gradually would be decreasing, leading to an unchangeable Dirac point.

S6. Photocurrent of the device

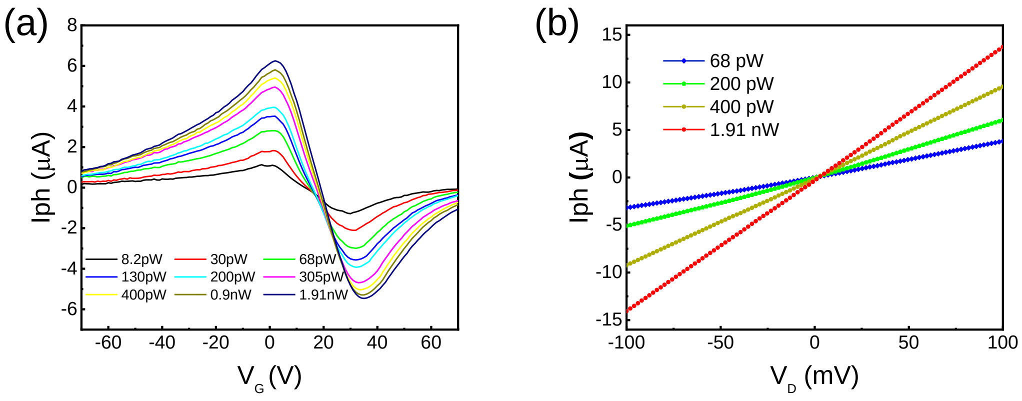

Figure S6. (a) The photocurrent ($I_{ph} = |I_{light} - I_{dark}|$) is a function of the gate voltage (V_{DS}= 50 mV, λ= 658 nm). (b) Calculated photocurrent (I_{ph}) of pentacene single crystal/graphene device as a function of the bias voltage (V_D).

S7. Universality of responsivity

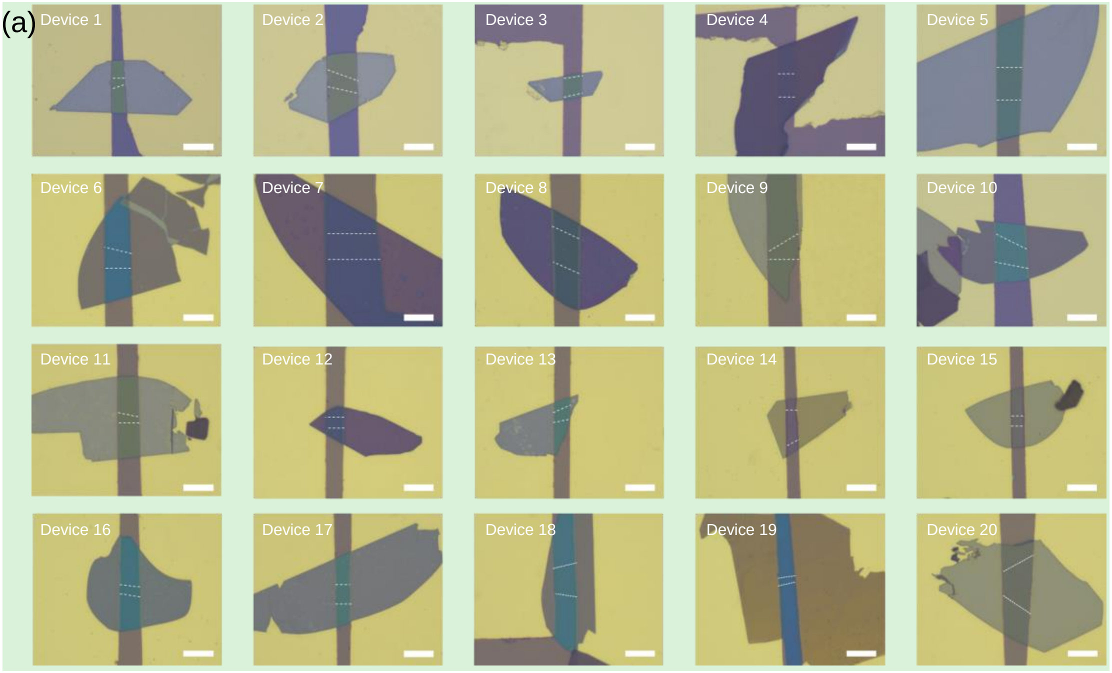

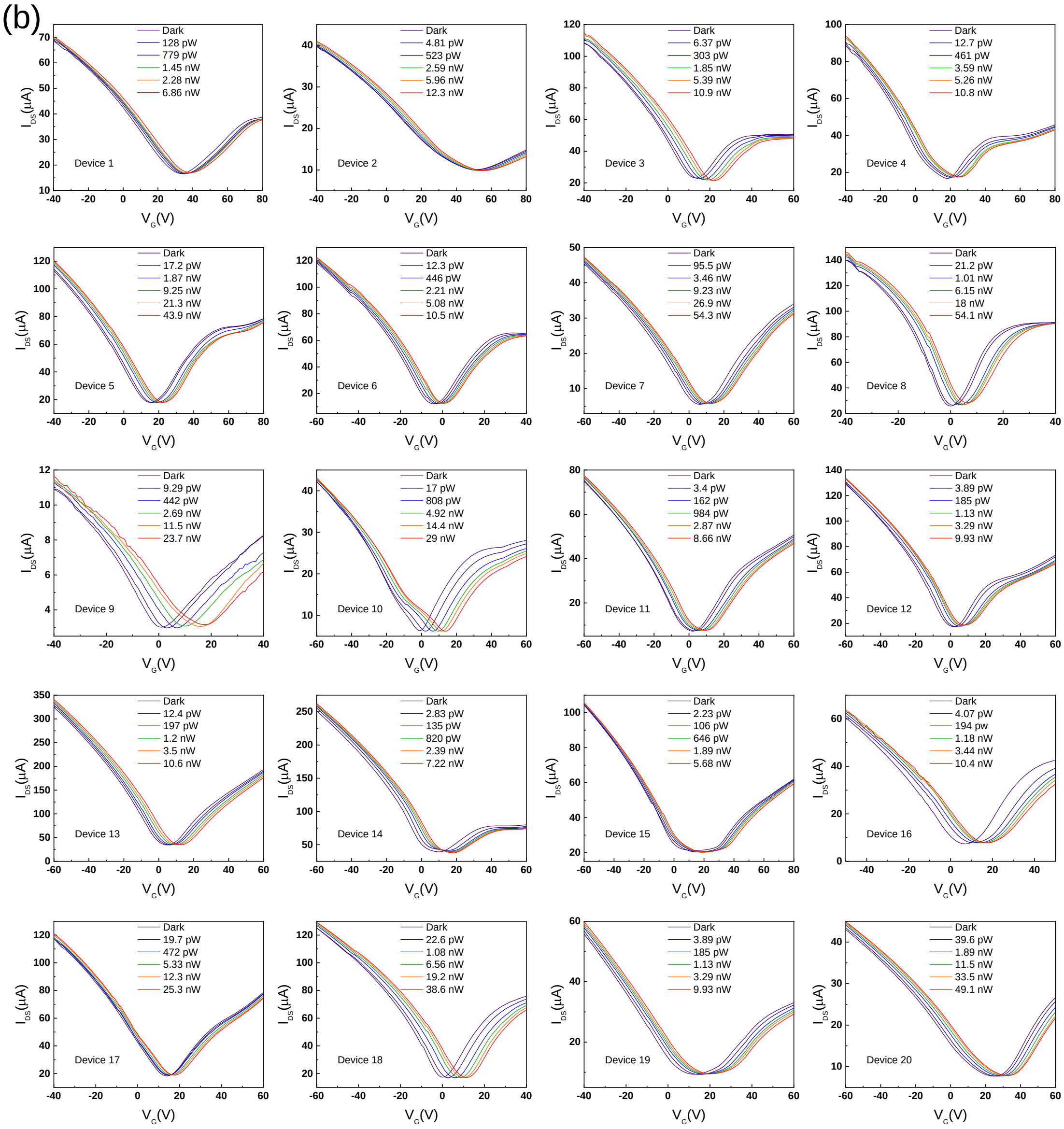

Figure S7. (a) Optical microscope images of other fabricated 20 hybrid devices. Scale bar: 20 μm. (b) Corresponding transfer curves of 20 devices under light illumination.

S8. KPFM images of Au/graphene

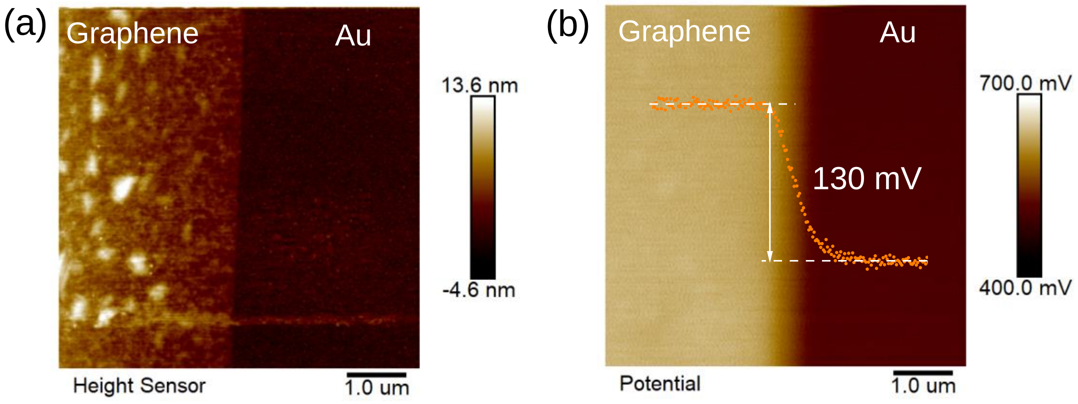

Figure S8. (a) AFM image of graphene sheet on gold film. (b) The corresponding KPFM image. Inset: surface potential profile.

S9. Photocurrent mapping at V_{DS}= 5 mV

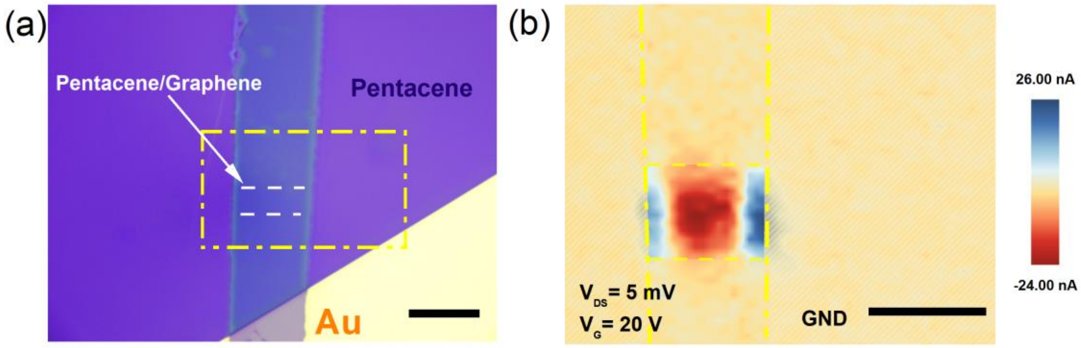

Figure S9. (a) Optical microscope image of the device. (b) Photocurrent mapping of conductive channel at low source drain voltage, scanning area is marked by yellow box in **a**. Scale bars: 15 μm.

S10. Photocurrent mapping of another device with asymmetric configration

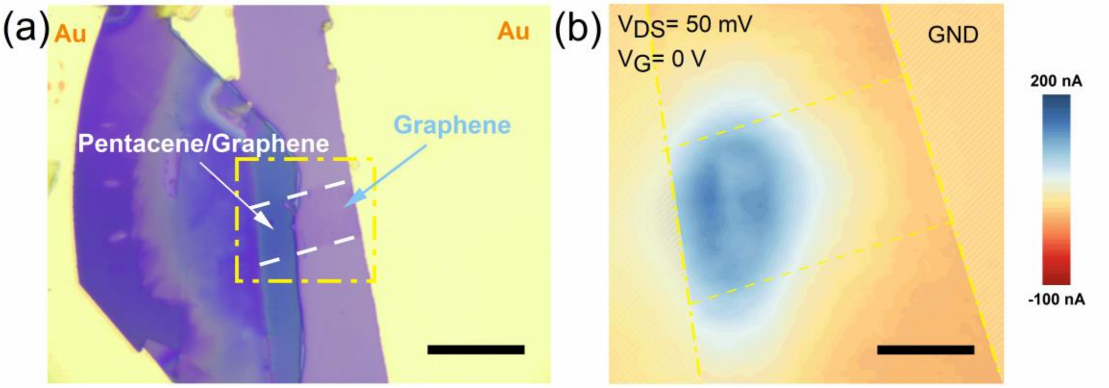

Figure S10. (a) Optical microscope image of the device. Scale bar: 15 µm. (b) Photocurrent mapping of conductive channel at V_{DS} = 50 mV, scanning area is marked by yellow box in **a**. Scale bar: 5 µm.

S11. Durability of the device

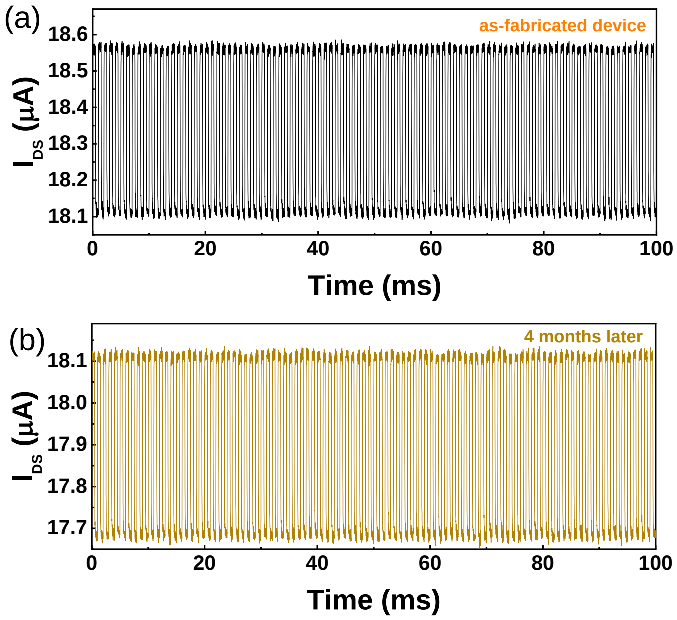

Figure S11. (a) Photocurrent response of the as-fabricated device is tested for the first time. (b) Photocurrent response of the device after 4 months without nitrogen protection.

S12. External quantum efficiency

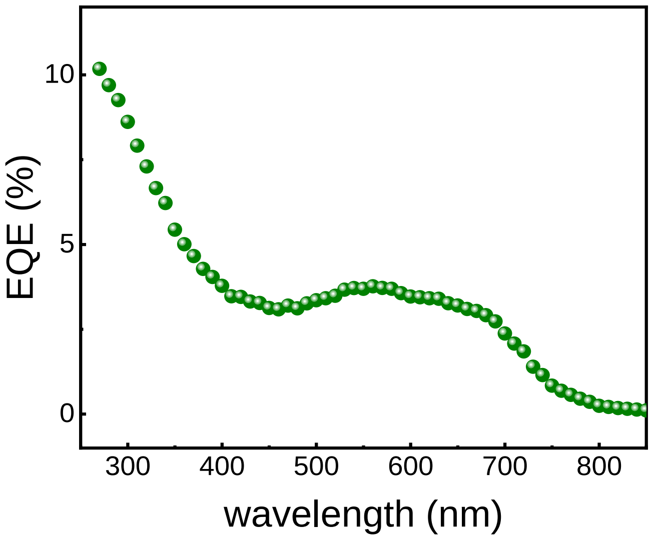

Figure S12. The quantum efficiency of graphene/pentacene device from 270 to 850 nm.
